# Successful on-ECLS Repair of CDH and Omphalocele in a Newborn

**DOI:** 10.1055/s-0043-1767734

**Published:** 2023-04-10

**Authors:** Frank Fideler, Migdad Mustafi, Hans-Joachim Kirschner, Ines Gerbig, Jörg Fuchs, Michael Hofbeck, Matthias Kumpf, Oliver Kagan, Jörg Michel, Walter Jost, Felix Neunhoeffer

**Affiliations:** 1 Department of Anesthesiology and Intensive Care Medicine, University Hospital, Tübingen, Germany; 2 Department of Thoracic, Heart and Vascular Surgery, University Hospital, Tübingen, Germany; 3 Department of Pediatric Surgery and Pediatric Urology, University Children's Hospital, Tübingen, Germany; 4 Department of Pediatric Cardiology, Pulmonology and Intensive Care Medicine, University Children's Hospital, Tübingen, Germany; 5 Department of Women's Health, University Women's Hospital, Tübingen, Germany; 6 Cardiovascular Engineering, University Hospital, Tübingen, Germany

**Keywords:** CDH and omphalocele, On-ECLS repair, newborn surgery

## Abstract

Both congenital diaphragmatic hernias (CDHs) and omphaloceles show relevant overall mortality rates as individual findings. The combination of the two has been described only sparsely in the literature and almost always with a fatal course. Here, we describe a term neonate with a rare high-risk constellation of left-sided CDH and a large omphalocele who was successfully treated on extracorporeal life support (ECLS). Prenatally, the patient was diagnosed with a large omphalocele and a left CDH with a lung volume of ∼27% and an observed to expected lung-to-head ratio of 30%. Due to respiratory insufficiency, an ECLS device was implanted. As weaning from ECLS was not foreseeable, the female infant underwent successful surgery on ECLS on the ninth day of life. Perioperative high-frequency oscillatory ventilation and circulatory and coagulation management under point-of-care monitoring were the main anesthesiological challenges. Over the following 3 days, ECLS weaning was successful, and the patient was extubated after another 43 days. Surgical treatment on ECLS can expand the spectrum of therapy in high-risk constellations if potential risks are minimized and there is close interdisciplinary cooperation.

## Introduction

Congenital diaphragmatic hernias (CDHs), as well as omphaloceles, can cause major problems for the treating team and are still associated with significant mortality. The overall mortality rate for omphalocele is 32.1%. 1 The CDH mortality rate hovers around 30%, and can rise to more than 60% after extracorporeal life support (ECLS) therapy in high-risk patients. 2 An observed to expected (o/e) lung-to-head ratio (LHR) <25%, liver herniation >20%, or o/e total fetal lung volume <25% are predictors of increased mortality and higher likelihood of requiring ECLS in CDH patients. 3

The combination of CDH and omphalocele presented here has an incidence of 1 to 2 per 100,000 live births based on the available literature. 4 Moreover, only a few publications can be found in which the patients also did not survive infancy.

In 2020, Mesas Burgos et al reported 36 patients with CDH and omphaloceles, of whom 14 patients survived. However, only 2 of these 14 survivors underwent ECLS and it is unclear if these patients were repaired during ECLS or after weaning. 5

This case report is one of the first to report on a congenital malformation consisting of left-sided CDH and a major omphalocele with pulmonary arterial hypertension (PAH) and their successful surgical management with ECLS therapy.

This approach is controversial in isolated CDH, but it can expand the treatment options for these patients and can contribute to increasing the chances of survival of these patients at suitable centers.

## Case Report

The present case report complies with the Equator Network CARE Checklist, version 2013. Written parental consent was obtained in advance.

**Fig. 1:**
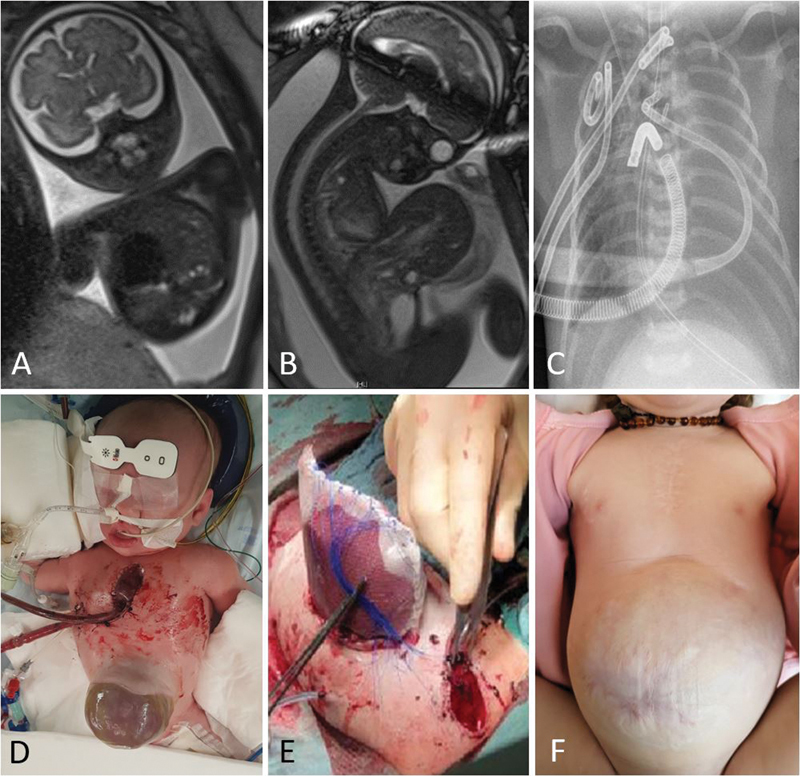
( **A** ) Magnetic resonance imaging (MRI) finding of left-sided congenital diaphragmatic hernia (CDH) at 33 weeks of gestation. ( **B** ) MRI finding of omphalocele at 33 weeks of gestation. ( **C** ) X-ray after cannulation for extracorporeal life support (ECLS). ( **D** ) Preoperative situs. ( **E** ) Situs after the operation. ( **F** ) Findings at 16 months of age.

The patient was diagnosed with a left-sided Bochdalek hernia, size type C, and omphalocele by magnetic resonance imaging (MRI) at week 32 of pregnancy. The left-sided lung could not be demarcated, and the lung volume on the right side was only 27% of the expected lung volume. The prenatal ultrasound revealed an o/e LHR of 30% and dextroposition of the heart. The liver was completely displaced into the hernial sac of the omphalocele, which was classified as a major omphalocele (diameter >4–5 cm; Fig. 1 ). 6

Cesarean section was performed before the onset of labor in the 38th week of gestation with Robson group 2b and intrauterine growth restriction. The birth weight was 2.9 kg. Apgar values of 1/5/10 and PH in combination with insufficient spontaneous breathing required high-frequency oscillatory (HFO) ventilation and inhaled nitric oxide after intubation. Structurally, the heart was unremarkable except for a dextroposition cordis, a persistent foramen ovale, and a tricuspid valve insufficiency. Milrinone, norepinephrine, and sildenafil therapy resulted in good biventricular function and stabilized PH with an X-shunt via the ductus arteriosus.

On the fifth day of life (DOL), there was lactic acidosis, a drop in preductal saturation below 85%, and increasing PH with a right to left shunt. Based on the recommendations of the CDH EURO Consortium, 7 ECLS was performed via a partial upper sternotomy. This approach allowed the greatest possible distance to be maintained from the omphalocele. The free life medical cannulas were placed in the ascending aorta (8 Fr) and the right atrium (12 Fr). The ECLS system had to be replaced on the eighth DOL due to clot activation and impending system occlusion despite rheoparin coating. As the patient's condition did not permit ECLS decannulation, it was decided by interdisciplinary consensus to perform surgery on the ninth DOL on ECLS due to the risk of further system occlusion.

In an operation time of 144 minutes, the CDH was approached via a transverse upper abdominal laparotomy. The stomach, spleen, and parts of the small and large intestines were found intrathoracically. The hernia was closed with an approximately 2× 3 cm large, 1-mm-thick Gore Dualmesh. The abdominal wall was temporarily closed with a silastic sheet (Schuster's plastic; Fig. 1 ).

In consideration of the perioperative bleeding risk and clot complications of ECLS, the running rate of unfractionated heparin was reduced and tranexamic acid was applied at the start of surgery. Intraoperative thrombelastographic point-of-care monitoring was used hourly and allowed statements on the plasmatic coagulation, the effect of heparin as well as the indication for treatment with fibrinogen or thrombocytes.

The established HFO ventilation, cardiac support therapy, and ECLS settings could be kept unchanged perioperatively.

In case of problems, there was an immediate exchange between surgeons, anesthetists, and perfusionists.

Transcranial sonography revealed no evidence of hemorrhage pre- or postoperatively.

ECLS weaning was performed over the following 3 days in the pediatric intensive care unit. PH persisted with half-system pressure in the right ventricle. Nineteen days later, an open fundoplication was performed, leaving the silastic sheet in place. After its removal, the abdominal wall was closed at the fascia level with a Vicryl mesh at the skin level. Temporarily Epigard and later a vacuum-assisted closure (VAC) therapy for 10 days led to spontaneous cutaneous healing. The patient was extubated on the 51st DOL. Parenteral feeding was started on the 10th DOL, and enteral nutrition was started on the 30th DOL. The girl was discharged home with a nasogastric feeding tube, sildenafil therapy, and minimal oxygen requirement.

Subsequently, at the age of 4 months, the child had to be hospitalized for 4 days because of a viral respiratory tract infection with an increased oxygen requirement. In the neuropediatric follow-up at the age of 6 months, a motor developmental delay with muscular hypotonia of the trunk and muscular hypertonia of the extremities was diagnosed.

Echocardiographic examination further revealed indirect signs of PH. For this, the girl is still in 3- to 6-month follow-ups.

## Discussion

Patients with CDHs show a high predisposition to PH due to pulmonary hypoplasia. 8 PH must also be expected in omphalocele cases in up to 57% of patients and concomitant cardiac disease in 20% of patients. 9 It does not seem suitable to assess the severity of the individual finding by bowel, stomach, or liver components that may now be located in one hernial cavity or the other. CDH staging according to the CDH Study Group does not allow conclusions to be drawn about group or stage survival in the presence of a concurrent omphalocele and was therefore not performed in our case. 10 Whether the combination of CDH and omphalocele further increases the risk for PH and its severity is unclear. However, due to the o/e total fetal lung volume, a complicated clinical course was to be expected, so that an adapted, findings-based procedure for all steps of therapy was agreed upon with the parents. 11

Therefore, currently, the treatment goals, possibility, and timing of surgical care must be set individually with close involvement of the parents.

For patients with isolated CDH and low and intermediate risk, surgical care planning on ECLS seems to outweigh the potential benefits due to potential complications. 12 With regard to survival 13 and bleeding complications 14 in low- and intermediate-risk patients, repair after ECLS weaning has been proven to be beneficial.

However, Jancelewicz et al reported a lower mortality with ECLS for high-risk patients (64.2 vs. 84.4%). 12

This was confirmed by several single-institution studies that suggested that early repair on ECLS may also be advantageous. 15 16 However, a recent study by Stewart et al in newborns with ECLS showed that 70% of CDH neonates experienced complications during their ECLS run, with the most common categories being metabolic (48.1%) and mechanical (38.9%), followed by hemorrhage (22.2%), neurological (18.5%), renal (11.1%), pulmonary (7.4%), and cardiovascular (7.4%). The median number of complications per patient was higher in the nonsurvivor group. In addition, mechanical (57.1 vs. 27.3%, *p* = 0.045) and renal (28.6 vs. 0%, *p* = 0.002) complications were more common among nonsurvivors than survivors. 17

Nevertheless, if ECLS weaning is not possible, early repair during ECLS in combination with lung-protective ventilation strategies and aggressive management of PH in extremely compromised neonates who otherwise would die is associated with good early survival. 18 Ideally, the patient should undergo on-ECLS repair within 72 hours of cannulation, which has been associated with improved survival rate, less bleeding, a shorter ECLS duration, and fewer circuit changes compared with the corresponding outcomes of late on-ECLS repair. 14 Anticoagulation protocols, including the perioperative administration of aminocaproic or tranexamic acid, and close perioperative monitoring of coagulation parameters have been associated with reduced bleeding risk in patients receiving on-ECLS repairs. 14 A specific perioperative hemostatic treatment enabled Keijzer et al to perform on-ECLS repair with a low frequency of bleeding complications, thereby taking advantage of having the physiologic benefits of ECLS available perioperatively. 19

In our case, the decisive factor for this approach was that using a sophisticated perioperative hemostatic treatment enables CDH repair on ECLS with a low frequency of bleeding complications. Early correction of the mechanical contributors to pulmonary and vascular pathophysiology while on ECLS may facilitate subsequent weaning in patients with a severe CDH phenotype and therefore afford a survival advantage. 19 20

The prerequisite for this therapy, analogous to the recommendations of the CDH EURO Consortium 7 for the surgical treatment of CDH, was a stable circulatory and oxygenation situation. Physiological ECLS effects, such as unloading of the right ventricle, may be a helpful therapeutic option postoperatively when pulmonary arterial crises occur.

For a complex subset of pediatric cardiac patients, ECLS has been shown to achieve improved results. 21

The long-term outcome of the child described herein remains to be seen.

## Conclusion

For the surgical on-ECLS treatment of CDH that is also in combination with other malformations, ECLS currently remains a case-by-case decision in high-risk constellations. However, based on the increasing experience with ECLS and positive individual case reports, ECLS seems suitable to become an integral part of the therapeutic spectrum for high-risk constellations in newborns in the future.

## Notes

New Insights and Importance for a Pediatric Surgeon The combination of congenital diaphragmatic hernia and omphalocele has been described only sparsely in the literature and almost always with a fatal course. The reported surgical treatment on ECLS can expand the spectrum of therapy in high-risk constellations if potential risks are minimized and there is close interdisciplinary cooperation.
